# Brain activity in the right-frontal pole and lateral occipital cortex predicts successful post-operatory outcome after surgery for anterior glenoumeral instability

**DOI:** 10.1038/s41598-017-00518-9

**Published:** 2017-03-29

**Authors:** Davide Zanchi, Gregory Cunningham, Alexandre Lädermann, Mehmet Ozturk, Pierre Hoffmeyer, Sven Haller

**Affiliations:** 1 Department of Psychiatry (UPK), University of Basel, Basel, Switzerland; 2 Division of Orthopaedic and Trauma Surgery, Department of Surgery, University Hospitals of Geneva, Geneva, Switzerland; 3 Faculty of Medicine of the University of Geneva, Geneva, Switzerland; 4 Division of Orthopaedic and Trauma Surgery, La Tour Hospital, Geneva, Switzerland; 5 Affidea Carouge Radiologic Diagnostic Center, Geneva, Switzerland; 6 Department of Surgical Sciences, Radiology, Uppsala University, Uppsala, Sweden; 7 Department of Neuroradiology, University Hospital Freiburg, Freiburg, Germany

## Abstract

Shoulder apprehension is more complex than a pure mechanical problem of the shoulder, creating a scar at the brain level that prevents the performance of specific movements. Surgery corrects for shoulder instability at the physical level, but a re-dislocation within the first year is rather common. Predicting which patient will be likely to have re-dislocation is therefore crucial. We hypothesized that the assessment of neural activity at baseline and follow-up is the key factor to predict the post-operatory outcome. 13 patients with shoulder apprehension (30.03 ± 7.64 years) underwent clinical and fMRI examination before and one year after surgery for shoulder dislocation contrasting apprehension cue videos and control videos. Data analyses included task-related general linear model (GLM) and correlations imaging results with clinical scores. Clinical examination showed decreased pain and increased shoulder functions for post-op vs. pre-op. Coherently, GLM results show decreased activation of the left pre-motor cortex for post-surgery vs. pre-surgery. Right-frontal pole and right-occipital cortex activity predicts good recovery of shoulder function measured by STT. Our findings demonstrate that beside physical changes, changes at the brain level also occur one year after surgery. In particular, decreased activity in pre-motor and orbito-frontal cortex is key factor for a successful post-operatory outcome.

## Introduction

Combining modern advances in functional neuroimaging and shoulder surgery has highlighted and defined the complex neural mechanisms behind shoulder apprehension in patients with glenohumeral instability. Recent works have proven that this previously presumed peripheral pathology induces significant cerebral alterations, the complexity of which cannot be comprehensively measured by clinical scores^1, 2^. However, these results are based on a single observation in a preoperative cohort of unstable patients. The evolution of apprehension with time, and particularly after surgical stabilization, remains to be elucidated.

Persistent apprehension following shoulder stabilization is a common finding^3^, affecting up to 16% of postoperative patients^4^. Although various etiologies may be in cause, including local mechanical failure^5^ or peripheral neurologic dysfunction^6^, a persistent central neurologic dysfunction remains an unexplored and highly probable explanation.

The aim of the present study was thus to compare the cerebral mapping of shoulder apprehension in patients with glenohumeral instability before and one year after surgical stabilization and correlate them with clinical scores. The hypothesis was that assessing neural changes could help to determine the post-operatory outcome.

The study design was carried out as following: After screening, all patients presenting shoulder glenoumeral instability undergo a clinical assessment and fMRI examination twice: at baseline (T1, pre-surgery) and at follow-up (T2 one year after shoulder stabilization surgery). During fMRI examination self-made animation movies (10 seconds) were visually presented. They represented activities potentially triggering apprehension, such as putting the right shoulder at risk for antero-inferior dislocation or arming the shoulder with a javelin, quickly reaching backwards for a seatbelt, and so forth (created by C.G.)^2^.

## Results

### Clinical outcome and scores

**Figure 1:**
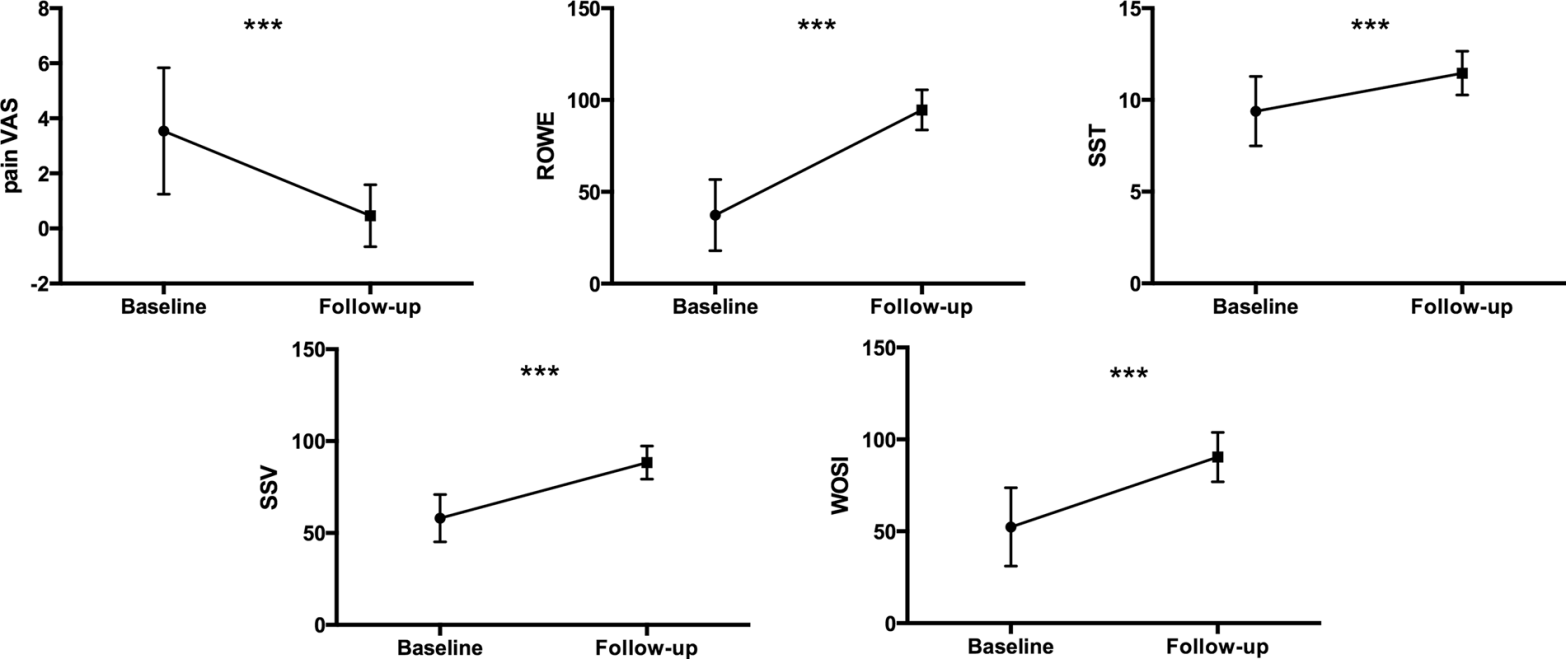
Paired t-test results. Significant differences were found in all the clinical scores at follow-up compared to baseline (p < 0.001). In particular considering ROWE, SST, SSV and WOSI scores an increased shoulder performance was found, while for pain VAS scores a decrease in pain levels were assessed. Mean and standard error were shown.

At one year (T2), no patients had sustained a re-dislocation or subluxation, and all were able to go back to their work and sporting activities. Paired t-test revealed significant differences (p < 0.001) in all the clinical scores between T1 and T2. In particular an increased shoulder performance was found for Rowe, Simple Shoulder Test (SST), Subjective Shoulder Value (SSV) and Western Ontario Shoulder Instability (WOSI), while decreased pain levels was found for pain Visual Analog Scale (pain VAS) (Fig. 1).

### Task-Related General Linear Model

**Figure 2:**
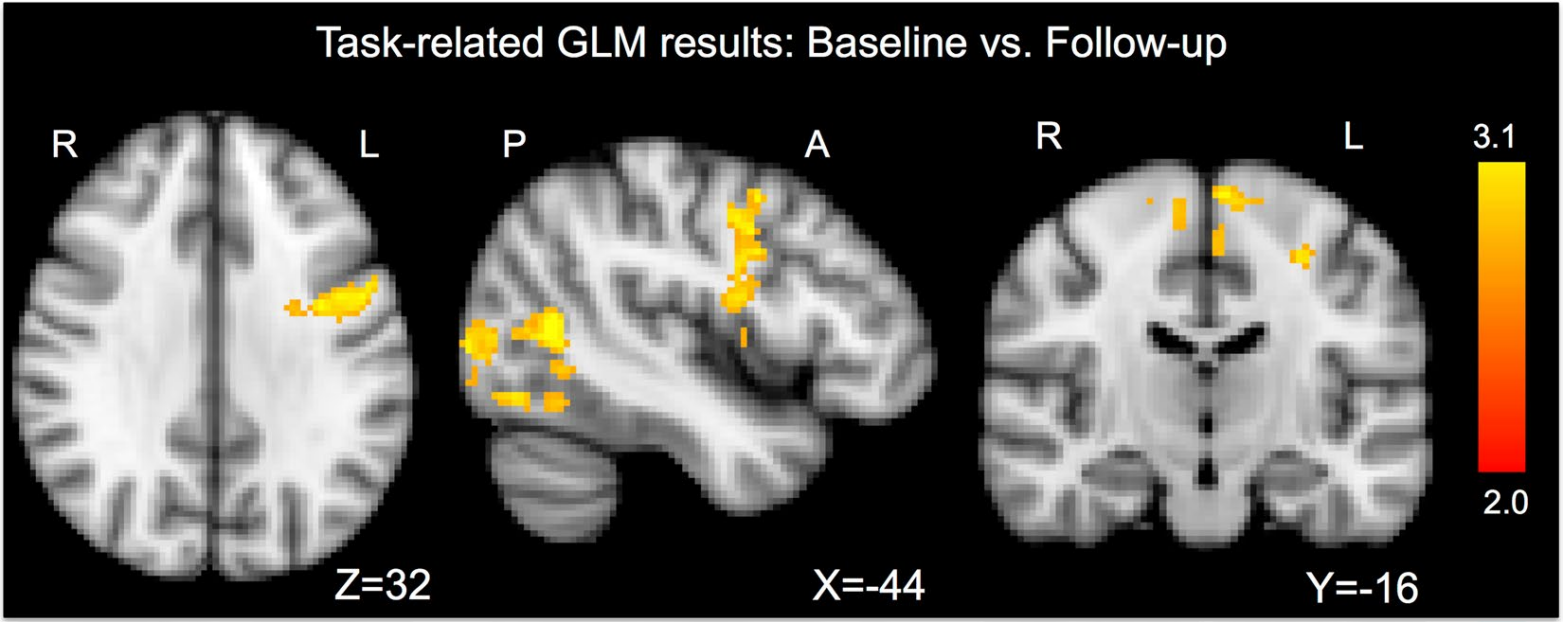
Task related GLM results.Task related GLM shows higher activation in Baseline vs. Follow-up for apprehension videos vs. control videos notably in the left motor and premotor cortex, somatosensory cortex, lateral occipital cortex, and para-hippocampal gyrus. Results show brain areas surviving TFCE correction. P values < 0.05 were considered as significant. No significant differences were found for the other comparisons.

Task related General Linear Model (GLM) shows higher activation in baseline vs. follow-up for apprehension videos vs. control videos notably in the left motor and premotor cortex, somatosensory cortex lateral occipital cortex and para-hippocampal gyrus (Fig. 2, Table 1A). No significant differences were found for the other comparisons.

**Table 1:** (A) shows the coordinates for the task-related GLM results. (B) shows the correlations between fMRI at T1 and SST scores at T2.

Cluster Index	Voxels	P	−log10(P)	Z-MAX	Z-MAX X (mm)	Z-MAX Y (mm)	Z-MAX Z (mm)	Side	Regions
**A) Task-related GLM results: Baseline vs. Follow-up.**									
1	3064	<0.001	7.22	3.06	46	−58	16	R	Lateral occipital cortex
2	863	<0.05	1.9	3.14	−44	−60	10	L	Middle termporal gyrus/Para-hippocampal gyrus
3	779	<0.05	1.63	2.97	−46	2	48	L	Pre-central gyrus
4	709	<0.05	1.39	3.04	16	−20	68	R	Post-central gyrus
**B) Correlations between fMRI at T1 and SST scores at T2**									
2	582	<0.05	1.77	2.97	56	−64	34	R	Frontal Pole
1	553	<0.05	1.63	3.24	40	60	−2	R	Occipital Cortex

Moreover, no significant results were found between left and right anterior glenohumeral instability patients for baseline vs. follow-up.

### Post-hoc GLM activation correlation with clinical scores

**Figure 3:**
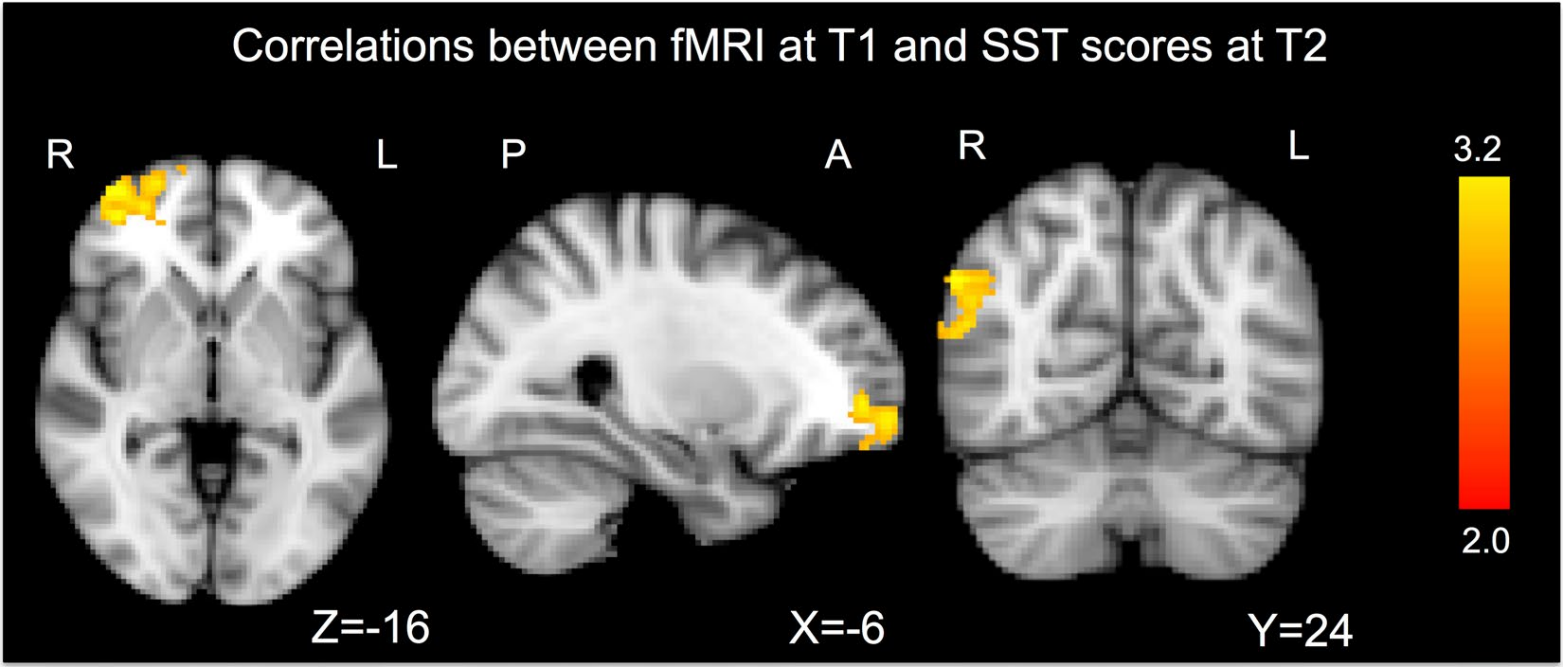
Post-hoc correlation analyses between the GLM activations at T1 and the clinical Simple Shoulder Test scores at T2. Higher activation in in the right-frontal pole and right occipital cortex correlates with SST scores at follow-up. Results show brain areas surviving TFCE correction. P values < 0.05 were considered as significant. No significant correlations were found for the other test scores.

From the post-hoc correlation analyses between the GLM activations at T1 and the clinical scores at T2, the SST yielded significant correlations. In particular SST scores positively correlated with activity in the right-frontal pole and in the right-occipital cortex. (Figure 3, Table 1B). No significant negative correlations were found between brain activations at T1 and SST scores at T1. No significant correlations were found for the other test scores.

### VBM and TBSS Analysis

The Voxel-based morphometry (VBM) analysis of gray matter (GM) density and the Tract-based spatial statistics (TBSS) analysis of white matter (WM) revealed no statistical differences between study groups.

## Discussion

The present study extends previous works, investigating brain changes before and after surgery for shoulder dislocation. Our findings demonstrate that stabilizing the shoulder induces changes at the brain level one year after surgery. Moreover, right–frontal pole (RFP) and right-occipital cortex activity is associated to a good outcome in shoulder performance. This is the first study that investigates brain changes after physical surgery and can be a model for future studies.

From an orthopedic point of view, apprehension is a common sign of anterior glenohumeral instability defined by fear of imminent dislocation elicited when bringing the arm to 90° of abduction and 90° of external rotation^1^. As previous studies demonstrate it is associated to several cognitive dimensions, as pain, anxiety and decreased shoulder functions^2^. Our first results relate to changes in clinical scores assessing different dimensions of shoulder apprehension one year after surgery for anterior shoulder dislocation. In particular, after surgery, an expected decrease in shoulder pain (pain VAS) and in parallel an increase in shoulder performance, stability and motility (SSV, SST, ROWE and WOSI) was found. These results are in line with previous studies and demonstrate an improvement in shoulder functions and decrease in pain after surgery^7–9^ confirming surgery as valid intervention for shoulder dislocation in properly selected patients^10^.

Our second and key finding shows that surgery for shoulder instability has also an effect on the brain, changing the response of specific regions to shoulder apprehension related stimuli, in our case video cues. In particular, our GLM results reveal higher activation of the pre-motor cortex before surgery compared to after surgery. These results are analogous to the findings of our previous work^1^, in which patients with shoulder instability were compared to healthy controls. Brain network connectivity was enhanced in patients with shoulder apprehension. The present longitudinal study confirms the previous findings showing frontal areas activity as reduced in patients after surgery for shoulder stabilization.

The pre-motor cortex is a brain area that is anterior the primary motor cortex and part of the middle/superior frontal gyrus that projects directly to the spinal cord and subsequently to the muscles, playing a role in the direct control of behavior^11, 12^. Our previous works demonstrated the involvement of this area in shoulder apprehension, showing higher activity as a sign of decrease in shoulder functionality^1^. The present results demonstrate that surgery for shoulder instability leads to a decrease of the activity in this cerebral area, suggesting that recovery also occurs at the brain level leading to functional neuroplasticity.

Post-surgery neuroplasticity was already demonstrated after cochlear implantation and ocular diseases, showing functional reorganization of the auditory and visual cortexes in response to surgery^13, 14^. Our findings apply this process to the orthopedic field revealing functional plasticity in the pre-motor cortex also after surgery for shoulder instability. Moreover, the same functional plasticity effects were also found in pre-operative versus post-operative patients in the lateral occipital cortex and para-hippocampal gyrus. The lateral occipital cortex has a role in object recognition^15^ and moreover in multi-sensor integration^16^, while the para-hippocampal gyrus has been associated with higher cognitive processes, including visuo-spatial processing and episodic memory. Therefore we can interpret our findings hypothesizing that during fMRI examination while the patients were watching the videos, they remembered situations in which a movement of the shoulder was performed, thus they integrate different physical sensations and pain was experienced. Our results show that after surgery, activation in these areas decreases and the brain shows a recovery effect.

Finally, our last results are highly clinically significant: we found that improvement (T2) of SST scores correlate with activation in the right frontal pole (RFP) and in the left occipital cortex. SST score measures shoulder functions in daily life^17^. Therefore, our findings demonstrate increased shoulder function as correlated to an activation in the RFP. We can conclude that activity in the frontal cortex is associated with better recovery after surgery for shoulder instability.

In general, the prefrontal cortex is a brain area involved in cognitive tasks and in higher cognitive functions^18, 19^. Our findings suggest that after seeing potential apprehension situations, the RFP does a fast evaluation of the shoulder function and then it decides if the movement can be performed or if pain sensations have to be elicited^2^. The involvement of the RFP in internal status check and pain regulation is confirmed by previous studies highlighting the functions of this areas in physical and movement related-situations^20, 21^. Therefore we can conclude that subjects with higher activity in the RFP after seeing apprehension cues are the ones to have the better improvement after shoulder surgery and that activity in RFP is related to a positive outcome for shoulder functions.

We have to specify that even though our analyses didn’t show any lateralization at the brain level of shoulder glenohumeral instability, we think this may plausibly occurs. The relative small sample size of the present study doesn’t allow an extensive investigation of this phenomenon. Therefore we suggest other studies with a bigger sample size to investigate the presence of lateralization effects shoulder apprehension at the brain level of.

As previous papers demonstrated shoulder apprehension is not only related to shoulder instability but also has consequences at the brain level, creating a scar that prevents the performance of specific movements^1, 2, 22^. After surgery, successful correction of shoulder instability occurs both on a physical and on a cerebral level, leading to neural plasticity effects. In particular motor and frontal areas are responsible of the regulation of this process.

### Strengths and limitations

It is important to notice that limitations are present in our work. First, we had a relatively small group size. This can impact our results, in particular the ones related to the lateralization at the brain level. We recommend studies with a bigger sample size to investigate the link between affected shoulder and brain activity. This investigation can then be also enhanced modifying our video-cue paradigm and showing videos related to only one side of affected shoulder. This may strengthen the results. Because apprehension involves both lateralized and non-lateralized cerebral regions, patient group constitution had to be selective (same hand dominance). This study therefore did not include any left-handed patients, as there were too few cases. Nevertheless, we still observed highly significant brain network changes in activity after surgery. This in turn indicates a strong effect of shoulder apprehension on cerebral networks pre- and post-operatively. Second, our results can be intuitively applied to other orthopedic conditions such as knee, elbow or ankle instability and the hypothesis that apprehension-related modifications in cerebral neuronal networks are presumably generic and not limited only to shoulder apprehension should be tested. Unveiling those mechanisms may provide a whole new approach to their management, such as fear extinction or neurofeedback^23^.

## Conclusion

The present study extends previous works showing differences at the brain level between healthy subjects and patients with shoulder apprehension, investigating brain changes after surgery for shoulder dislocation. Our findings demonstrate that beside physical changes, changes at the brain level also occur one year after surgery. In particular, pre-motor and orbito-frontal cortex activity correlates with a good outcome in shoulder performance. This is the first study that investigates brain changes after physical surgery and can serve as a model for future studies investigating the interconnections of the body-brain matrix.

## Methods

### Participants

The present study was approved by the Ethics Committee of the University Hospitals of Geneva, Switzerland and conducted in accordance with the principles of the Declaration of Helsinki. All experimental procedures were carried out in accordance with the approved guidelines. Prior to participation, the subjects signed a written informed consent form. Inclusion criteria were male patients with traumatic unidirectional (anteroinferior) glenohumeral instability, a positive apprehension sign whether painful or not, and radiologic evidence of their instability (Bankart lesion, anterior glenoid bone loss, Hill-Sachs lesion). Exclusion criteria included (1) patients with pain at rest, (2) preoperatively radiological signs of dislocation arthropathy, (3) hyperlaxity defined as more than 85° of elbow-to-waist external rotation, (4) an abnormal visual acuity, (5) reported history of major medical disorders (cancer, cardiac illness), head injury, psychiatric or neurologic disorders, alcohol or drug abuse, and (6) participants who used psychotropics, stimulants, or beta-blockers on a regular basis. The final sample included 13 right-handed (10 with right-sided and 3 with left-sided glenohumeral instability) male patients with a mean age of 30.03 ± 7.64 years.

### Clinical scores assessment and study procedure

All patients underwent a clinical assessment twice: at baseline (T1, pre-surgery) and at follow-up (T2 one year after shoulder stabilization surgery). As several studies demonstrated, within the first year of follow-up a re-dislocation can occur^24^. A delay of one year before reassessment was chosen in order to allow a full recovery and the return to work and sport activities.

The clinical assessment included five commonly used subjective scores in the form of self- administered questionnaires (Table 1), prior to functional magnetic resonance (fMRI). The questionnaires were administered to investigated clinical dimensions associated to shoulder instability. The pain Visual Analog Scale (VAS)^25^ is a widely used single item score where the patient rates pain intensity between zero and ten. This scale is useful for patient pre- and postoperative monitoring^26^. The Simple Shoulder Test (SST)^17^ consists of twelve binary “yes” or “no” questions evaluating shoulder performance in daily activities. Subjective Shoulder Value (SSV) is a single question, where the patient is asked to rate his overall shoulder function as a percentage of a normal shoulder^27^. It is a quick and easily administered score that has also been validated for various shoulder disabilities, such as instability. The Rowe score for instability^28^ is a 3 item score with 4 choices each, measuring shoulder function, stability and motion. The final result is converted to a value between 0 and 100. Finally, the Western Ontario Shoulder Instability (WOSI)^29^ score is a 21 items score also specific for shoulder instability, measuring the degree of disability in activities of daily living. The final result is also converted to a value between 0 and 100. Except for pain VAS, higher results mean higher function.

### Surgical procedure

All surgeries were carried out by two specialized shoulder surgeons (GC and AL). Patients were positioned in a semi-beach chair position, under general anesthesia with an interscalenic block or catheter. Surgical procedures consisted in open or arthroscopic Bankart labral repairs or Latarjet-Patte bone block. Operations were carried according to standard described procedure using three 2.8 mm suture anchors for Bankart porcedures (N = 3), subscapularis split and triple-locking mechanism for open Latarjet (N = 6), and a modified 5 portal technique^22^ for arthroscopic Latarjet (N = 4). Postoperatively, patients were immobilized in a sling for 2 weeks after a Latarjet procedure and 6 weeks after a Bankart procedure. All went back to their previous level of work and sport activities after 3 months, and none re-dislocated before their T2 assessment at one year.

### fMRI Acquisition

Images were obtained using a 3 T scanner (Trio; Siemens, Erlangen, Germany) with a standard 32-channel head-coil. FMRI imaging of the whole brain was performed using echo planar imaging employing the following parameters: whole brain coverage, 96 × 96 matrix, TR = 2.5 s, TE = 30 ms, 39 slices, 148 repetitions. A 3D T1-weighted structural scan (256 × 256 matrix size, 176 sections, 1 × 1 × 1 mm^3^, TE = 2.3 ms, TR = 2300 ms) and a diffusion tensor imaging (DTI) scan (30 diffusion directions *b* = 1000 s/mm^2^ isotropically distributed on a sphere, 1 reference *b* = 0 s/mm^2^ image with no diffusion weighting, 128 × 64 matrix, 2 × 2 × 2 mm voxel size, TE = 92 ms,TR = 9000 ms, 1 average) were acquired.

### fMRI task

The paradigm was described and validated previously^1, 2^ and consisted of an on-off block-design with two active conditions (apprehension cue and control videos) and a rest condition. During the active condition video cues^2^ were used**;** these animation movies (10 seconds) showed daily activities that trigger shoulder apprehension. Control videos were matched for content except for the absence of cues inducing shoulder apprehension. After each video, a visual analog scale was presented for 2.5 seconds and participants rated the degree of perceived apprehension using an MRI-compatible response box. The rating scale included nine steps from no apprehension to high apprehension. After the rating, a rest period followed, including the visual presentation of a fixation cross for 17.5 seconds. Each participant performed two runs. Within each run, lasting for 370 seconds each, 6 apprehension and 6 control videos were shown in a pseudo-randomized fashion. Before MRI scanning, participants were familiarized with the procedure and performed a training run outside the scanner.

### Statistical Analysis

Statistical analyses were conducted using GraphPad Prism (Version 6, GraphPad Software, San Diego, USA) and FSL (Version 5.0.6, FMRIB, Oxford, UK).

### Analysis of Clinical and Demographic data

After performing D’Agostino-Pearson omnibus test to check for normal distribution, those variables that were normally distributed, notably pain VAS, ROWE, SSV, WOSI and SST scores, were submitted to a paired t-test to investigate differences in shoulder functions between baseline and follow up.

### Task-Related General Linear Model

Processing and analysis of imaging data was performed using FSL FEAT (FMRI Expert Analysis Tool version 6.00, http://fsl.fmrib.ox.ac.uk/fsl/fslwiki/FEAT). Preprocessing included brain extraction using FSL’s BET (Brain Extraction Tool), motion correction using FSL’s MCFLIRT (intra-modal motion correction tool)^30^ and smoothing of 5 mm using FSL’s SUSAN (noise reduction uses nonlinear filtering)^31^. The pre-processed functional images were first co-registered to the structural images and later on normalized to MNI space.

The linear-model analysis included three levels. At the first level, the contrast ‘apprehension videos versus control videos’ was calculated separately for each run of each participant using fixed-effects analysis. Then, at the second level, one fixed-effects analysis was conducted including both runs of each subject. Finally, at the third level, a paired t-test analysis was performed to investigate group differences between baseline and follow-up. This resulted in a mixed-effects group model implementing FLAME-1 (FMRIB’s Local Analysis of Mixed Effects). Finally, multiple comparisons correction by threshold-free cluster enhancement (TFCE)^32^ was applied. P values < 0.05 were considered as significant.

Furthermore, to test for lateralization effects another level of GLM analysis was performed. In particular, subjects with left and right-sided anterior glenohumeral instability at baseline vs. follow-up were contrasted. Once more, multiple comparisons correction by threshold-free cluster enhancement (TFCE)^32^ was applied. P values < 0.05 were considered as significant.

### Post-hoc GLM activation correlation with clinical scores

Correlations between fMRI results and clinical outcome at follow-up were investigated. After pre-processing, at the third level of the GLM, correlations analyses between the imaging results at T1 and the scores of the clinical tests (T2) (pain VAS, ROWE, SST, SSV and WOSI) were performed. The main predictor was the demeaned and normalized (values between −1 and 1) clinical score across all subjects. Finally, a correction for multiple comparisons by threshold-free cluster enhancement (TFCE) was applied. P values < 0.05 were considered as significant.

### VBM Analysis of T1 Images

To assess differences in gray matter density between groups, a voxel-based morphometry (VBM) analysis was performed in FSL (FSL Version 5.0.9; http://fsl.fmrib.ox.ac.uk), using standard processing steps^33, 34^. Firstly, BET extraction and tissue-type segmentation were performed using the corresponding FSL tools (Brain Extraction Tool and FAST4). Secondly, non-linear transformation into Montreal Neurological Institute (MNI) reference space was applied and a study-specific gray matter (GM) template was created. The native GM images were then non-linearly registered to this template. Finally, the images were smoothed with an isotropic Gaussian kernel of 2 mm sigma. A voxel-wise GLM was implemented using permutation-based nonparametric testing (Randomise, part of FSL). Age and gender were used as non-explanatory regressors. Results were corrected for multiple comparisons using TFCE^35^ and p values < 0.05 were considered as significant.

### Analyses of white matter structures

FSL software was used to analyze diffusion tensor imaging (DTI) data, according to the standard procedure^36^ to test for white matter (WM integrity differences between groups. First, by a non-linear registration all subjects’ fractional anisotropy data was projected onto a mean fractional anisotropy tract skeleton. Later, by using a non-linear registration, a voxel-wise GLM was implemented using permutation-based nonparametric testing (Randomise, part of FSL). Threshold free cluster enhancement correction for multiple comparisons was performed, considering TFCE corrected P < 0.05 as significant.

## References

[1] Cunningham G (2015). Neural Correlates of Clinical Scores in Patients with Anterior Shoulder Apprehension. Med. Sci. Sports Exerc.

[2] Haller S (2014). Shoulder apprehension impacts large-scale functional brain networks. AJNR Am. J. Neuroradiol..

[3] Lädermann A (2013). Risk factors for dislocation arthropathy after Latarjet procedure: a long-term study. Int. Orthop..

[4] Ropars, M. *et al.* Diagnosis and treatment of anteroinferior capsular redundancy associated with anterior shoulder instability using an open Latarjet procedure and capsulorrhaphy. *Knee Surg. Sports Traumatol. Arthrosc. Off. J. ESSKA* (2015). doi:10.1007/s00167-015-3621-9. 10.1007/s00167-015-3621-9 26003480

[5] Lädermann, A. *et al.* Does surgery for instability of the shoulder truly stabilize the glenohumeral joint? *Medicine* (*Baltimore*) **95**, (2016). 10.1097/MD.0000000000004369 PMC4979797 27495043

[6] Myers, J. B. & Lephart, S. M. Sensorimotor deficits contributing to glenohumeral instability. *Clin. Orthop.* 98–104 (2002). 10.1097/00003086-200207000-00013 12072751

[7] Bedi A, Ryu RKN (2009). The treatment of primary anterior shoulder dislocations. Instr. Course Lect..

[8] Arciero RA, St Pierre P (1995). Acute shoulder dislocation. Indications and techniques for operative management. Clin. Sports Med..

[9] Longo UG (2014). Management of primary acute anterior shoulder dislocation: systematic review and quantitative synthesis of the literature. Arthrosc. J. Arthrosc. Relat. Surg. Off. Publ. Arthrosc. Assoc. N. Am. Int. Arthrosc. Assoc.

[10] Brophy RH, Marx RG (2009). The treatment of traumatic anterior instability of the shoulder: nonoperative and surgical treatment. Arthrosc. J. Arthrosc. Relat. Surg. Off. Publ. Arthrosc. Assoc. N. Am. Int. Arthrosc. Assoc.

[11] Rushworth MFS, Johansen-Berg H, Göbel SM, Devlin JT (2003). The left parietal and premotor cortices: motor attention and selection. NeuroImage.

[12] Dum RP, Strick PL (1991). The origin of corticospinal projections from the premotor areas in the frontal lobe. J. Neurosci. Off. J. Soc. Neurosci..

[13] Martins Rosa A, Silva MF, Ferreira S, Murta J, Castelo-Branco M (2013). Plasticity in the human visual cortex: an ophthalmology-based perspective. BioMed Res. Int.

[14] Fallon JB, Irvine DRF, Shepherd RK (2008). Cochlear implants and brain plasticity. Hear. Res..

[15] Grill-Spector K, Kourtzi Z, Kanwisher N (2001). The lateral occipital complex and its role in object recognition. Vision Res..

[16] Beauchamp MS (2005). See me, hear me, touch me: multisensory integration in lateral occipital-temporal cortex. Curr. Opin. Neurobiol..

[17] Lippitt, S. B. *et al.* A practical tool for evaluating function: the Simple Shoulder Test. (1993).

[18] Miller EK (2000). The prefrontal cortex and cognitive control. Nat. Rev. Neurosci..

[19] Miller EK, Cohen JD (2001). An integrative theory of prefrontal cortex function. Annu. Rev. Neurosci..

[20] Winston JS, Vlaev I, Seymour B, Chater N, Dolan RJ (2014). Relative Valuation of Pain in Human Orbitofrontal Cortex. J. Neurosci..

[21] Strigo IA, Spadoni AD, Lohr J, Simmons AN (2014). Too hard to control: compromised pain anticipation and modulation in mild traumatic brain injury. Transl. Psychiatry.

[22] Cunningham G, Benchouk S, Kherad O, Lädermann A (2016). Comparison of arthroscopic and open Latarjet with a learning curve analysis. Knee Surg. Sports Traumatol. Arthrosc. Off. J. ESSKA.

[23] Kubik A, Biedroń A (2013). Neurofeedback therapy in patients with acute and chronic pain syndromes–literature review and own experience. Przegla̧d Lek..

[24] Shibata H (2014). Risk factors for shoulder re-dislocation after arthroscopic Bankart repair. J. Orthop. Surg.

[25] Huskisson EC (1982). Measurement of pain. J. Rheumatol..

[26] Martinez-Urrutia A (1975). Anxiety and pain in surgical patients. J. Consult. Clin. Psychol..

[27] Gilbart MK, Gerber C (2007). Comparison of the subjective shoulder value and the Constant score. J. Shoulder Elb. Surg. Am. Shoulder Elb. Surg. Al.

[28] Rowe CR, Patel D, Southmayd WW (1978). The Bankart procedure: a long-term end-result study. J. Bone Joint Surg. Am.

[29] Kirkley A, Griffin S, McLintock H, Ng L (1998). The development and evaluation of a disease-specific quality of life measurement tool for shoulder instability. The Western Ontario Shoulder Instability Index (WOSI). Am. J. Sports Med..

[30] Jenkinson M, Bannister P, Brady M, Smith S (2002). Improved optimization for the robust and accurate linear registration and motion correction of brain images. NeuroImage.

[31] Brady, J. M., S. S. SUSAN - a new approach to low level image processing. *Int. J. Comput. Vis.* (1997).

[32] Winkler AM, Ridgway GR, Webster MA, Smith SM, Nichols TE (2014). Permutation inference for the general linear model. NeuroImage.

[33] Smith SM (2006). Tract-based spatial statistics: voxelwise analysis of multi-subject diffusion data. NeuroImage.

[34] Smith SM (2007). Acquisition and voxelwise analysis of multi-subject diffusion data with tract-based spatial statistics. Nat. Protoc..

[35] Smith SM, Nichols TE (2009). Threshold-free cluster enhancement: addressing problems of smoothing, threshold dependence and localisation in cluster inference. NeuroImage.

[36] Smith SM (2004). Advances in functional and structural MR image analysis and implementation as FSL. NeuroImage.

